# Interdisciplinary reconstructions of the forearm and hand

**DOI:** 10.1515/iss-2025-0025

**Published:** 2025-11-27

**Authors:** Luca Kümmerl, Theresa Promny, Elisabeth Eschenbacher, Andreas Arkudas, Raymund E. Horch

**Affiliations:** Department of Plastic and Hand Surgery, University Hospital of Erlangen, Friedrich-Alexander University of Erlangen-Nuernberg, Erlangen, Germany

**Keywords:** upper limb reconstruction, interdisciplinary surgery, hand trauma, reconstructive surgery, plastic surgery

## Abstract

**Objectives:**

This study examines the clinical application of interdisciplinary reconstructive strategies for complex forearm and hand defects. Emphasis is placed on principles guiding flap selection, anatomical localization, and the central role of plastic surgeons within coordinated surgical teams. The aim is to clarify how collaborative planning and technique adaptation influence reconstructive outcomes in upper extremity salvage.

**Methods:**

A retrospective review was conducted of 79 patients who underwent upper extremity reconstruction between 2020 and 2024 at a tertiary care center. Inclusion required composite defects of the hand or forearm treated with local, regional, or free flaps. Demographics, etiology, defect location, reconstructive technique, interdisciplinary collaboration, and complications were analyzed descriptively.

**Results:**

Most patients were male (n=62), with a mean age of 53.3 years. Defects most often involved the long fingers (n=32) and thumb (n=31). Trauma (68 %) was the main cause, followed by oncologic resection (22 %) and infection (10 %). Free flaps (n=15) were primarily used for larger or composite defects requiring microsurgical reconstruction, while local and regional flaps were employed zone-specifically for smaller soft tissue defects, especially in digits and thumb. Functional reconstruction addressed tendon, nerve, vessel, and bone injuries. The overall complication rate was 12.6 %, with no flap failures or limb loss.

**Conclusions:**

Upper limb reconstruction benefits from individualized, anatomy-driven planning and interdisciplinary cooperation. Plastic surgeons play a key role in adapting techniques to anatomical zones, achieving favorable functional and aesthetic outcomes.

## Introduction

Complex defects of the upper extremity pose a significant challenge in modern reconstructive surgery due to the intricate anatomy and functional importance of the hand and forearm. These regions are composed of layered soft tissue envelopes, highly specialized tendons, dense neurovascular structures, and important articulations between multiple bones that collectively facilitate fine motor skills, grip strength, and tactile sensibility. As a result, injuries affecting the upper limb—whether traumatic, oncologic, or infectious origin—often lead to substantial disability if not managed properly.

Successful reconstruction of upper limb defects requires more than anatomical closure; it demands the restoration of motion, stability, and sensation. This has given rise to increasingly individualized surgical concepts, particularly in the realm of plastic surgery, where microsurgical techniques, functional tendon and nerve transfers, and an understanding of vascular territories support the approach to reconstruction. Traditionally, the reconstructive ladder—starting from secondary intention healing, progressing through skin grafts, and ending in free tissue transfer—served as the conceptual basis for procedural selection. However, this model is gradually being supplanted by more dynamic frameworks like the reconstructive elevator and matrix, which permit the surgeon to escalate or de-escalate treatment complexity based on defect characteristics and patient-specific variables [1], [2], [3], [4].

Advancements in perforator flap anatomy, composite tissue engineering, and the standardization of interdisciplinary case planning have further enhanced outcomes. Prior studies have reinforced the importance of early, definitive soft tissue coverage and cross-specialty coordination in improving salvage rates, reducing donor site morbidity, and minimizing the need for secondary revisions [2], 5].

This study evaluates interdisciplinary approaches to upper limb reconstruction in a tertiary setting, focusing on the interdisciplinary role of plastic surgery in managing complex defects of the forearm and hand. We present a retrospective evaluation of patient characteristics, surgical techniques, and complication profiles across a broad spectrum of etiologies. This study is designed to contribute evidence toward refining reconstructive decision making in the upper limb, with implications for both surgical safety and functional outcome. The reconstructive approaches undertaken for a large cohort of patients with upper limb defects at a tertiary academic center. Furthermore, the diversity of reconstructive techniques used, ranging from local advancement flaps to microsurgical free tissue transfer, offers insight into the evolving landscape of limb salvage and functional restoration in upper extremity reconstruction.

Complex injuries and defects of the forearm and hand, resulting from trauma, infection, or oncologic resection, often require advanced reconstructive strategies. Plastic surgeons play a central role within interdisciplinary teams, using the reconstructive ladder as a guiding framework to restore both function and form.

## Methods

### Study design and ethical approval

This retrospective study was methodologically structured to capture the complexity and variability of upper limb reconstruction as practiced in a high-volume tertiary care center. All patient data were anonymized in compliance with ethical standards and data protection regulations. The retrospective character of this study is in accordance with the institutional ethics committee and the Helsinki Declaration and its later amendments or comparable ethical standards.

### Patient selection

All patients undergoing reconstructive surgery for complex soft tissue or composite defects of the forearm and hand between January 2020 and December 2024 were eligible for inclusion. Inclusion criteria were presence of composite tissue loss involving skin, subcutaneous tissue, tendon, nerve, vessel, or bone and treatment involving local, regional, and free flaps. Patients with superficial soft tissue defects suitable to conservative treatment or primary closure were excluded.

### Data acquisition

Electronic patient records, operative reports, and interdisciplinary surgical planning documents were reviewed. Demographic variables included age, sex, and laterality of the defect. Operative parameters captured included type of reconstructive technique (e.g., local flap, regional flap, free flap), flap subtype, operating time, and intraoperative collaboration. Complication data included partial or complete flap loss, infection, wound dehiscence, and donor-site morbidity. All patients had a minimum follow-up period of 6 months.

### Surgical technique and interdisciplinary approach

All patients underwent preoperative planning in interdisciplinary conferences involving plastic surgeons, orthopedic trauma or oncologic surgeons and vascular surgeons when indicated, anesthesiologists, and physiotherapists. Surgical strategies were defined jointly and adapted according to defect size, exposed structures, vascular status, and donor site availability. In complex reconstructions, procedures were performed collaboratively by multidisciplinary teams to ensure optimal intraoperative coordination between reconstructive and orthopedic or oncologic surgery. Pre- and perioperative imaging was planned in close consultation with radiologists to evaluate vascular anatomy, soft tissue conditions, and implant positioning, thereby supporting surgical planning and timing. Postoperative care included standardized rehabilitation protocols.

### Statistical analysis

Descriptive statistics were used to summarize demographic and surgical variables. Categorical data were presented as frequencies and percentages, while continuous data were expressed as means and ranges. Complication rates were calculated and subgrouped by technique to evaluate relative procedural risk.

## Results/case presentations

A total of 79 patients who underwent upper limb reconstruction between 2020 and 2024 were included in this retrospective analysis. Patient characteristics are summarized in Table 1. The mean age was 53.3 years (range 16–85 years), with 62 male and 17 female patients. The laterality of the defects was nearly evenly distributed, with 40 affecting the right and 39 the left upper extremity. Mean operative time across all procedures was 148.3 min (range 30–714 min), reflecting a broad spectrum of case complexity. Etiologically, trauma was the predominant indication for reconstruction (n=54), followed by oncologic resections (n=17) and infections (n=8). Traumatic causes included complex wounds and amputations. The oncologic group consisted mainly of malignant melanoma and squamous cell carcinoma, while infections were primarily soft tissue infections and wrist empyema. Defect localization analysis revealed that 30 defects involved the long fingers (index to little finger), and 28 were localized to the thumb. The remaining defects affected the forearm (n=12), wrist (n=2), and hand (n=7). The reconstructive approach was tailored to the anatomical zone and functional demands of each defect.

**Table 1:** Patient characteristics, defect location, type of reconstruction, and complications.

Total patients	79
Mean age [years] (range)	53.3 (16–85)
Sex (male/female)	62/17
Side of defect (right/left)	40/39
Mean operative time [minutes] (range)	148.3 (30–714)
**Etiology, n (%)**	
Trauma	54 (68.4)
Oncologic	17 (21.5)
Infection	8 (10.1)
**Defect location, n (%)**	
Long fingers	30 (38.0)
Thumb	28 (35.4)
Hand	7 (8.9)
Wrist	2 (2.5)
Forearm	12 (15.2)
**Flap type, n (%)**	
Free flaps (total)	15 (19.0)
Pedicled/local flaps (total)	64 (81.0)
**Functional reconstructions** (no. of patients)	
Bone	14
Tendon	9
Vessel	9
Nerve	6
**Total complications, n (%)**	10 (12.6)
Wound dehiscence	3
Excessive flap volume	2
Reinfection	1
Partial flap necrosis	1
Donor site complication	2
Tumor recurrence	1

**Figure 1:**
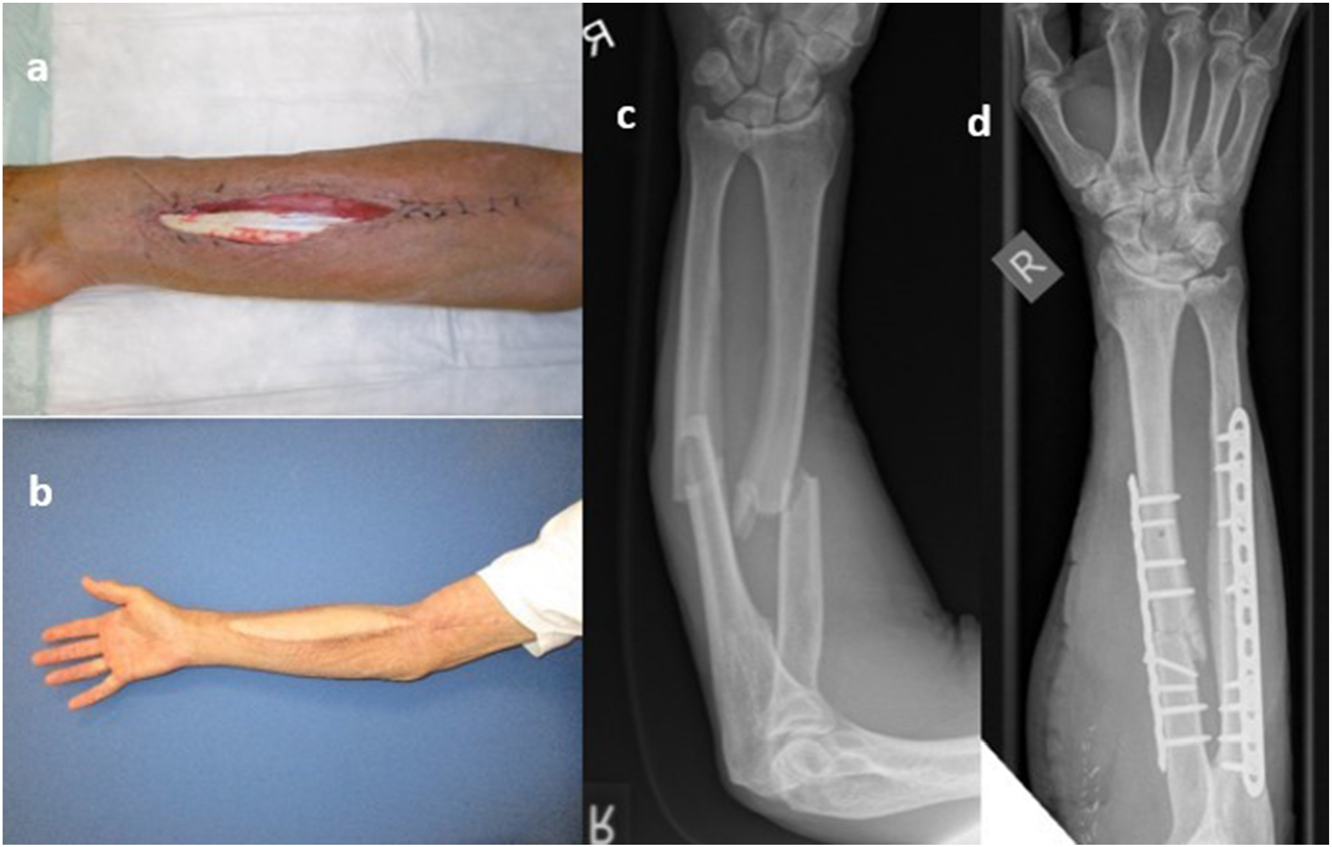
Male patient with soft tissue defect of the right forearm following open forearm fracture (a). (b) Two months postoperatively after defect coverage using a free anterolateral thigh (ALT) flap. (c)/(d) Pre- and postoperative X-rays of the right forearm.

Reconstructive strategy followed established reconstructive principles, allowing for case-specific technique selection (Figure 1). Free flap reconstruction was performed in 15 cases: eight anterolateral thigh (ALT) flaps, three vertical rectus abdominis myocutaneous (VRAM) flaps, one latissimus dorsi flap, one temporalis fascia flap, and one gracilis flap. Pedicled flaps were more commonly employed: 14 patients received Foucher flaps (or modifications thereof) [6], 12 Moberg advancement flaps [7] were applied for volar thumb defects, and 8 Keystone flaps were used for larger soft tissue defects. Cross-finger and reversed cross-finger flaps were employed in 29 cases, primarily for fingertip and larger finger defects. One patient was reconstructed with a dorsal metacarpal artery (DMCA) flap. The choice of flap closely reflected defect localization: Cross-finger flaps were primarily used for long finger pulp and dorsal defects; Moberg flaps were reserved for volar thumb reconstructions; Foucher flaps and their modifications were used for more extensive defects of the thumb. For larger or composite defects involving the wrist or forearm, Keystone and free flaps (ALT, VRAM) were the reconstructive method of choice.

Involvement of functional anatomical structures varied. Tendon reconstruction was performed in 9 cases, vascular repair in 9, bone stabilization in 14, and nerve coaptation in six.

Complications occurred in 10 patients (12.6 %). All of them were manageable through secondary interventions. One patient, initially reconstructed due to a primary infection, required reoperation following reinfection, highlighting the need for vigilant postoperative monitoring in infection-associated cases. Wound dehiscence was observed in three patients and treated with minor revision. Two patients required secondary flap thinning after pedicled flap transfer to the thumb due to excess bulk impairing fine motor control. These were addressed electively to improve dexterity and functional contour. One case of partial distal flap necrosis occurred after pedicled reconstruction of the dorsal hand; the area healed successfully following minor surgical revision. No partial flap necrosis was observed in free flap reconstructions, and no complete flap loss occurred in any patient treated with either free or pedicled flap reconstruction. In donor site–related complications included a single dehiscence at the anterolateral thigh harvest site and one superficial wound infection, both resolved with conservative measures or limited surgical intervention. An additional complication was noted in one oncologic patient who required reoperation for a local tumor recurrence within the previously reconstructed field. Importantly, there were no total flap failures or secondary limb losses documented throughout the cohort.

### Case 1

A 71-year-old male patient sustained a closed, displaced fracture of the radial and ulnar shafts of the right forearm following a carriage accident. Emergency fracture stabilization was performed at an outside hospital using dual plate osteosynthesis. Postoperatively, the patient developed a hematoma at the surgical site. Five days later, hematoma evacuation was performed through the palmar-radial incision used for the initial fracture fixation. Due to marked soft tissue swelling, primary wound closure was not feasible.

The patient was subsequently transferred to our institution. To optimize wound conditions and manage the soft tissue swelling, a negative pressure wound therapy system was applied and changed twice repeatedly. Preoperative vascular imaging with digital subtraction angiography (DSA) and phlebography demonstrated intact vascular structures in the proximal right forearm. Ten days after admission, with persistent soft tissue defect and exposed tendons, defect coverage was achieved using a contralateral ALT free flap. Microvascular anastomosis was performed in an end-to-side fashion to the brachial artery, while venous outflow was established with two venous couplers applied to accompanying veins.

The flap remained viable throughout the postoperative period without signs of vascular compromise. Wound healing was uneventful, providing stable and durable soft tissue coverage of the forearm. The donor site also healed without complications, and the patient was discharged in good general condition with preserved function of the affected limb (Figure 1).

### Case 2

A 61-year-old male patient presented to our plastic and hand surgery clinic with a history of tumor resection at the volar aspect of the left wrist performed abroad approximately 2 years ago. Histopathological analysis had revealed a malignant peripheral nerve sheath tumor. Adjuvant radiotherapy was performed.

Over the following months, the patient developed a recurrent mass at the volar wrist. MRI demonstrated an ill-defined, hyperintense lesion. Multidisciplinary tumor board discussion at our institution recommended surgical resection.

Wide local excision was performed, including resection of the palmaris longus tendon, epineural tissue of the median nerve, and the ulnar artery. Negative pressure wound therapy was applied. Subsequent procedures included further resections involving the flexor carpi radialis tendon and the flexor pollicis longus tendon in three additional operations. Final histopathological analysis with molecular testing confirmed a clear cell sarcoma of tendons and aponeuroses, resected with negative margins (R0).

**Figure 2:**
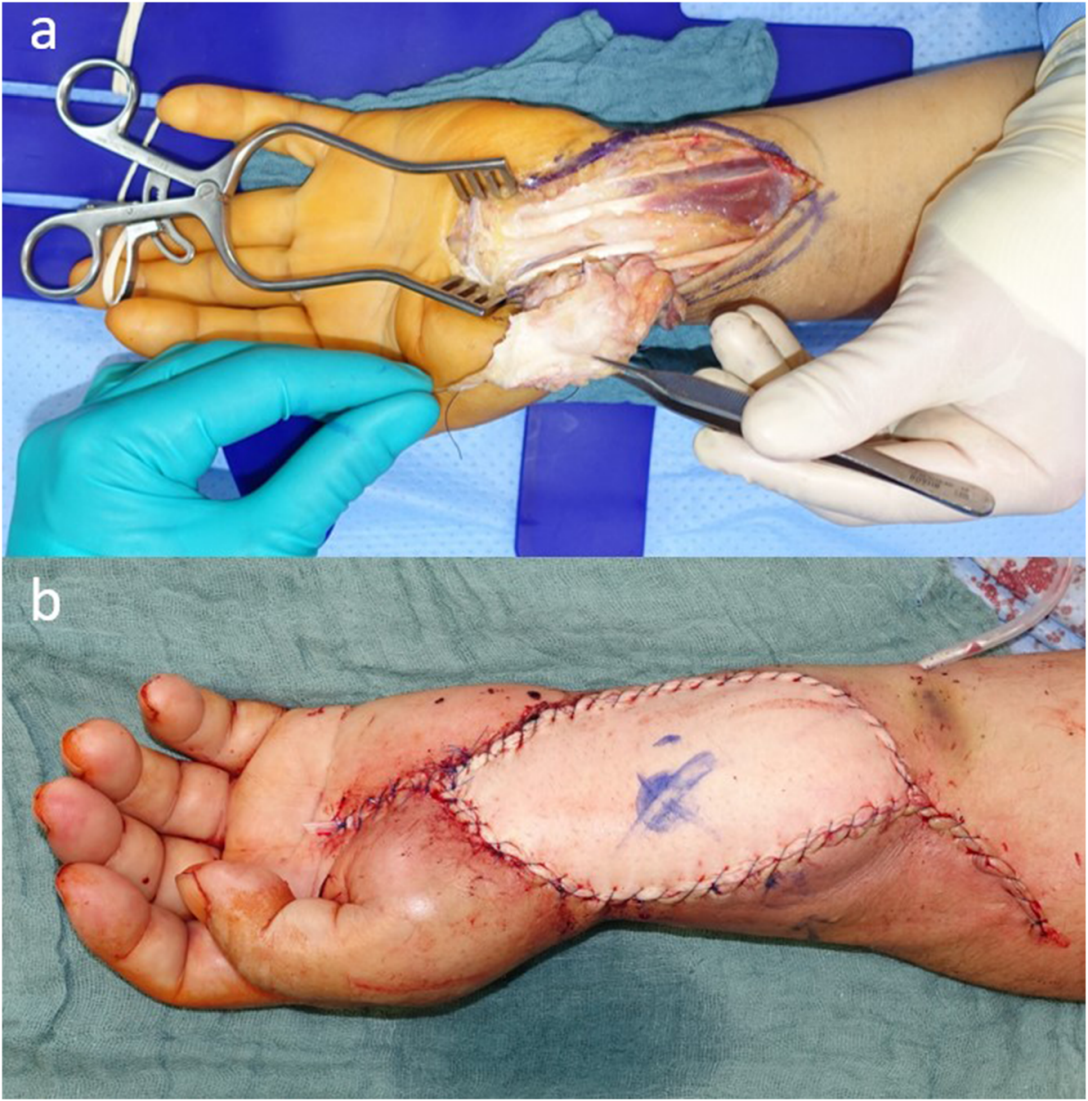
Male patient with soft tissue defect of the left forearm following tumor resection (a) and after defect coverage using a free anterolateral thigh (ALT) flap (b).

Functional reconstruction was achieved after R0-resection by transfer of the flexor digitorum superficialis tendon of the ring finger. In addition, vascular reconstruction of the left ulnar artery was performed using a venous interposition graft harvested from the ipsilateral forearm. Definitive defect coverage was achieved with a free contralateral ALT flap. Microvascular anastomosis was performed in an end-to-side fashion to the radial artery, while venous outflow was established using two venous couplers to accompanying veins (Figure 2).

The case was reviewed postoperatively multiple times in the multidisciplinary tumor board, where the option of reirradiation was discussed but not recommended due to prior radiotherapy. The patient was enrolled in close follow-up with regular local MRI surveillance and systemic staging. While no local recurrence was detected, follow-up CT raised suspicion of pulmonary metastasis in the right lung, which was scheduled for surgical resection by thoracic surgery.

### Case 3

A 41-year-old male patient sustained a severe saw injury to the right middle finger, resulting in an open multifragmentary middle phalanx fracture and complete laceration of the extensor tendon in zone II with segmental tissue loss, as well as an open end phalanx fracture of the ring finger. Emergency surgery was performed on the day of injury, including K-wire osteosynthesis and reconstruction of the lateral bands of the extensor mechanism using a palmaris longus tendon graft harvested from the forearm.

**Figure 3:**
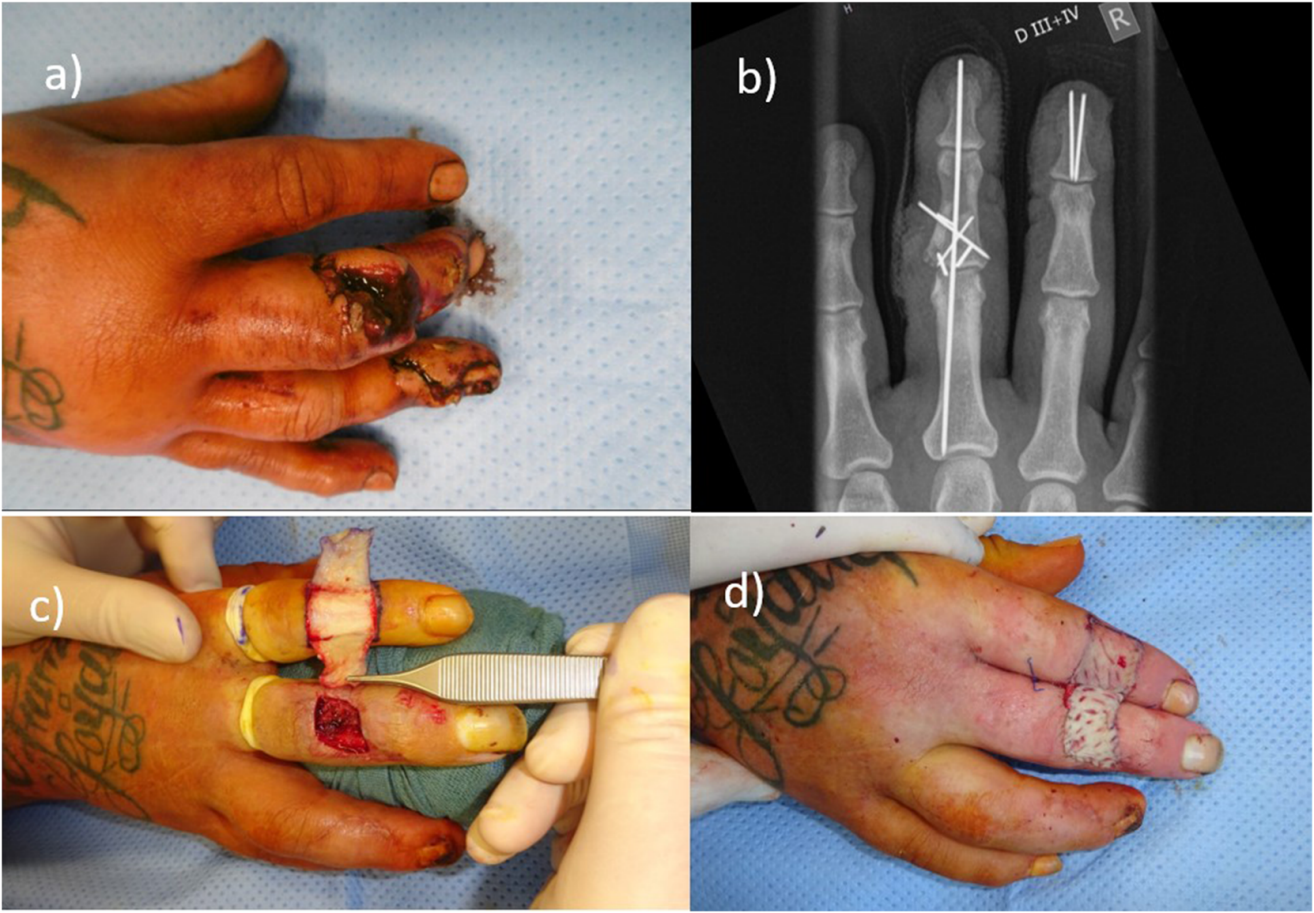
Male patient with saw injury of the right middle and ring finger (a). (b) Postoperative X-ray after K-wire osteosynthesis of the right middle and ring finger. (c)/(d) Soft tissue coverage of the right middle finger with reversed cross-finger flap from the index finger.

At 6 weeks postoperatively, the K-wires were removed following radiographic confirmation of bony consolidation, and active mobilization therapy was initiated. Subsequently, the patient developed a wound-healing complication on the dorsal aspect of the middle phalanx of the middle finger. Repeated surgical debridements were required, followed by definitive soft tissue coverage with a reversed cross-finger flap from the dorsal middle phalanx of the index finger and a full-thickness skin graft harvested from the forearm (Figure 3).

Three weeks later, flap division was performed, and the postoperative course was then uneventful. Intensive physiotherapy and occupational therapy were required postoperatively to achieve a satisfactory range of motion, followed by several weeks of continued rehabilitation therapy to optimize functional recovery.

Together, these results and case presentations highlight the specific, interdisciplinary application of a broad range of reconstructive techniques, demonstrating the real-world utility of the reconstructive elevator concept in upper limb salvage.

## Discussion

This study confirms the essential role of plastic surgery in managing complex defects of the forearm and hand within an interdisciplinary framework. Our findings underscore the increasing relevance of the reconstructive elevator model [8], 9], enabling early, safe, and functionally driven decision making. The high proportion of local and regional flaps reflects a stratified approach depending on defect severity and anatomical location. The selective use of free flaps such as the ALT, VRAM, and latissimus dorsi underlines their role as reliable, first-line options for extensive or composite tissue defects [10], 11].

Importantly, close interdisciplinary planning and intraoperative collaboration between plastic, trauma, orthopedic, vascular, and hand surgeons proved crucial for addressing bony stabilization (18 %), vascular reconstruction (4 %), tendon transfer (11 %), and nerve repair (8 %), supporting findings from others on the importance of collaborative strategies [12], [13], [14]. Beyond the operative phase, continuous coordination with anesthesiology, radiology, and physiotherapy teams was key to ensuring optimized timing, vascular monitoring, and functional rehabilitation. This multidisciplinary workflow allows early detection of complications and targeted therapy adjustments, directly influencing outcomes and patient satisfaction.

Our data further illustrate that even with a predominance of trauma-related injuries (68 %), tailored reconstructive solutions—ranging from perforator-based designs [15] to free flaps [16]—can achieve favorable outcomes with low complication rates. The observed 12.7 % complication rate was manageable and did not result in any flap failures or limb loss.

To optimize flap viability and prevent postoperative complications, indocyanine green (ICG) fluorescence angiography has emerged as a valuable intraoperative tool. By allowing real-time visualization of tissue perfusion, ICG can significantly reduce the incidence of flap necrosis, particularly in borderline-perfused or irradiated tissue beds. Its utility has been well documented in both perforator and free flap surgery [17], 18]. Future reconstructive algorithms may benefit from its routine incorporation into flap monitoring and inset assessment.

In cases of absent or damaged recipient vessels—common in post-traumatic defects—arteriovenous (AV) loops offer an effective solution to establish reliable microsurgical anastomosis sites. AV-loop creation, typically via saphenous vein grafts, has been shown to extend the indication for free flap reconstruction even in anatomically compromised regions [19].

Furthermore, in extensive trauma or oncologic resections where vascular anatomy is unclear, preoperative imaging via computed tomographic angiography (CTA), magnetic resonance angiography (MRA), or DSA should be considered standard. These modalities enhance preoperative planning by accurately identifying recipient vessels, guiding pedicle length, and avoiding intraoperative surprises. Their role in extremity reconstruction has been validated in several studies demonstrating improved flap safety and reduced intraoperative uncertainty [20], [21], [22]. Particularly in free flap cases with high complexity, preoperative vascular mapping contributes to shorter operative time and improved safety.

A two-stage reconstructive approach can be particularly beneficial in cases of extensive trauma, infection, or oncologic resection. In the first stage, thorough debridement and temporary wound conditioning using negative-pressure wound therapy (NPWT) promote granulation tissue formation, reduce edema and bacterial load, and allow clear demarcation of nonviable tissue. This interim phase facilitates optimal preparation of the wound bed for subsequent coverage. In the second stage, definitive reconstruction—whether local, pedicled, or free flap—is performed under improved and controlled conditions. Moreover, in oncologic settings, this strategy allows bridging of the interval until final histopathological margin confirmation, ensuring safe and timely reconstruction. The benefits of NPWT in optimizing local perfusion, decreasing wound complications, and improving flap success have been well documented [23].

Local flaps like cross-finger and Moberg flaps remain essential in fingertip and pulp reconstructions due to their reliability, ease of execution, and minimal donor site morbidity. Recent long-term outcome data support the clinical durability and functional success of cross-finger flap reconstruction in digital defect coverage, with low complication rates and favorable sensory outcomes [24]. Keystone perforator island flaps further expand options for intermediate-size defects, particularly in elderly or comorbid patients where free flap surgery may pose elevated risk [25], 26].

Ultimately, the reconstructive elevator allows the surgeon to bypass rigid stepwise escalation and instead select the technique best suited to functional needs, anatomical constraints, and patient-specific factors. In this context, interdisciplinary coordination—from initial debridement and microsurgical planning to physiotherapy and occupational reintegration—forms the cornerstone of modern reconstructive success in upper limb salvage.

The retrospective design inherently limits the ability to establish causal relationships and introduces potential selection bias. Moreover, while short- to mid-term outcomes including flap viability and complication profiles were systematically recorded, long-term functional results were not consistently available. Future prospective studies should, therefore, incorporate standardized functional outcome assessments and long-term follow-up protocols to better clarify the true impact of reconstructive strategies on quality of life and hand function.

## Conclusions

This study highlights the effectiveness of different reconstructive strategies to approach upper limb reconstruction. Through integration of plastic, orthopedic, vascular, and rehabilitative expertise, interdisciplinary planning proved essential for achieving safe, functional, and zone-specific reconstructions. The case-based application of local, regional, and free flap techniques enabled tailored solutions across a heterogeneous patient cohort. Plastic surgeons served a central role in coordinating complex reconstructions, particularly in high-demand zones such as the thumb, forearm, and multidigit injuries. High salvage rates, low complication profiles, and avoidance of limb loss underscore the clinical relevance of an individualized and collaborative reconstructive strategy.

These findings affirm that structured interdisciplinary coordination and flexible reconstructive algorithms are key components of successful management in upper extremity reconstruction.
